# An Introductory Tutorial on Cardiovascular Pharmacogenetics for Healthcare Providers

**DOI:** 10.1002/cpt.2957

**Published:** 2023-06-12

**Authors:** Akinyemi Oni-Orisan, Sony Tuteja, Glenda Hoffecker, D. Max Smith, Matteo Castrichini, Kristine R. Crews, William A. Murphy, Nam H. K. Nguyen, Yimei Huang, Christelle Lteif, Kevin A. Friede, Kelan Tantisira, Folefac Aminkeng, Deepak Voora, Larisa H. Cavallari, Michelle Whirl-Carrillo, Julio D. Duarte, Jasmine A. Luzum

**Affiliations:** 1 Department of Clinical Pharmacy, University of California San Francisco, San Francisco, California, USA;; 2 Division of Translational Medicine and Human Genetics, Department of Medicine, Perelman School of Medicine, University of Pennsylvania, Philadelphia, Pennsylvania, USA;; 3 MedStar Health, Columbia, Maryland, USA;; 4 Department of Oncology, Georgetown University Medical Center, Washington, DC, USA;; 5 Department of Cardiovascular Medicine, Mayo Clinic, Rochester, Minnesota, USA;; 6 Department of Pharmacy and Pharmaceutical Sciences, St. Jude Children’s Research Hospital, Memphis, Tennessee, USA;; 7 Division of Pharmacotherapy and Experimental Therapeutics, UNC Eshelman School of Pharmacy, University of North Carolina at Chapel Hill, Chapel Hill, North Carolina, USA;; 8 Department of Pharmacotherapy and Translational Research and Center for Pharmacogenomics and Precision Medicine, University of Florida, Gainesville, Florida, USA;; 9 Division of Cardiology, University of North Carolina School of Medicine, Chapel Hill, North Carolina, USA;; 10 Division of Respiratory Medicine, Department of Pediatrics, University of California San Diego, San Diego, California, USA;; 11 Departments of Medicine and Biomedical Informatics (DBMI), Yong Loo Lin School of Medicine, National University of Singapore, Singapore City, Singapore;; 12 Centre for Precision Health (CPH), National University Health System (NUHS), Singapore City, Singapore;; 13 Precision Medicine Program, Department of Medicine, Duke University School of Medicine, Durham, North Carolina, USA;; 14 Department of Biomedical Data Science, Stanford University, Stanford, California, USA;; 15 Department of Clinical Pharmacy, University of Michigan College of Pharmacy, Ann Arbor, Michigan, USA;; 16 Center for Individualized and Genomic Medicine Research, Henry Ford Health System, Detroit, Michigan, USA.

## Abstract

Pharmacogenetics can improve clinical outcomes by reducing adverse drug effects and enhancing therapeutic efficacy for commonly used drugs that treat a wide range of cardiovascular diseases. One of the major barriers to the clinical implementation of cardiovascular pharmacogenetics is limited education on this field for current healthcare providers and students. The abundance of pharmacogenetic literature underscores its promise, but it can also be challenging to learn such a wealth of information. Moreover, current clinical recommendations for cardiovascular pharmacogenetics can be confusing because they are outdated, incomplete, or inconsistent. A myriad of misconceptions about the promise and feasibility of cardiovascular pharmacogenetics among healthcare providers also has halted clinical implementation. Therefore, the main goal of this tutorial is to provide introductory education on the use of cardiovascular pharmacogenetics in clinical practice. The target audience is any healthcare provider (or student) with patients that use or have indications for cardiovascular drugs. This tutorial is organized into the following 6 steps: (1) understand basic concepts in pharmacogenetics; (2) gain foundational knowledge of cardiovascular pharmacogenetics; (3) learn the different organizations that release cardiovascular pharmacogenetic guidelines and recommendations; (4) know the current cardiovascular drugs/drug classes to focus on clinically and the supporting evidence; (5) discuss an example patient case of cardiovascular pharmacogenetics; and (6) develop an appreciation for emerging areas in cardiovascular pharmacogenetics. Ultimately, improved education among healthcare providers on cardiovascular pharmacogenetics will lead to a greater understanding for its potential in improving outcomes for a leading cause of morbidity and mortality.

Pharmacogenetics is a relatively new field of pharmacology that can significantly improve clinical outcomes through reduced adverse drug effects and enhanced efficacy for drugs that are commonly used to treat a wide range of cardiovascular diseases. Unfortunately, most current healthcare providers have very little, if any, education on cardiovascular pharmacogenetics. For example, only 29% of physicians have had any pharmacogenetic training in medical school or post-graduation, and only 10% of physicians feel adequately informed about pharmacogenetic testing.^1^ Moreover, current health professional school curricula have very little, if any, education on cardiovascular pharmacogenetics.^2,3^ For example, 77% of pharmacy schools believe that pharmacists do not have the appropriate knowledge of pharmacogenetics, but only 31% indicated that their programs planned to expand pharmacogenetics in their curriculum.^3^ Even a brief amount of education on cardiovascular pharmacogenetics has been shown to significantly improve healthcare providers’ attitudes toward pharmacogenetics. For example, a 1-hour grand rounds seminar significantly improved physicians’ attitudes toward cardiovascular pharmacogenetics.^4^ Therefore, the main goal of this tutorial is to provide introductory education on the use of cardiovascular pharmacogenetics in clinical practice. The target audience is current healthcare providers (or students) with patients that use or have indications for cardiovascular drugs, for example, physicians (both generalists and specialists), pharmacists, and nurses. This tutorial is organized in the 6 following steps: (1) understand basic concepts in pharmacogenetics; (2) gain foundational knowledge of cardiovascular pharmacogenetics; (3) learn the different organizations that release cardiovascular pharmacogenetic guidelines and recommendations; (4) know the current cardiovascular drugs to focus on clinically and the supporting evidence; (5) discuss an example patient case of cardiovascular pharmacogenetics; and (6) develop an appreciation for emerging areas in cardiovascular pharmacogenetics. This tutorial was developed by members of the Pharmacogenomics Global Research Network (PGRN; https://pgrn.org/), which is a global leader in pharmacogenetic research and education since 2000, when it began as the National Institutes of Health (NIH)-funded Pharmacogenetic Research Network. Additional resources for healthcare providers on genomic and pharmacogenomic education can be found online through the National Human Genome Research Institute (https://www.genome.gov/For-Health-Professionals/Provider-Genomics-Education-Resources) and the Pharmacogenomics Education Program (PharmGenEd; http://pharmacoge/nomics.ucsd.edu/). Readers are referred elsewhere for the following topics that are beyond the scope of this tutorial: cost-effectiveness of pharmacogenetics^5–7^; the process for the clinical implementation of pharmacogenetics (https://www.pharm/gkb.org/page/pgxImplementationResources); whether or not to order a pharmacogenetic test (as opposed to what clinical actions can be taken when pharmacogenetic information is already available)^8^; and clinical infrastructure/information technology issues relevant to pharmacogenetics.^9,10^

## STEP 1: UNDERSTAND BASIC CONCEPTS IN PHARMACOGENETICS

The safety and efficacy of a particular drug can vary substantially from person to person. Several known factors that can influence how a patient responds to a drug include their age, weight, sex, comorbidities, renal and hepatic function, diet, and drug–drug interactions. An additional, and more recently recognized factor is genetics. The terms pharmacogenetics and pharmacogenomics refer to the field of research and clinical practice focused on how genetic factors influence drug response. The terms pharmacogenetics and pharmacogenomics are typically used interchangeably, but pharmacogenetics usually refers to an individual or only a few genes from the genome, and pharmacogenomics usually refers to the entire genome. The total amount of variation in drug response that can be explained by genetics, also known as heritability, is quite wide and depends on the specific drug and the drug response phenotype assessed.^11^ For example, based on a study of mono- and dizygotic twins, 91% of the heritability of metoprolol and 86% of torsemide pharmacokinetics can be explained by genetic factors.^12^ From a large study of statin users that also included a subset of first-degree relatives, the heritability of statin low-density lipoprotein cholesterol (LDL-C) response was estimated at 12%.^13^ Therefore, patients’ genetics play an important role, and sometimes the majority role, in their response to drugs, and healthcare providers should have at least a basic understanding of pharmacogenetics.

Patients can inherit genetic variants that result in changes to the function or expression of the protein products that are important for drug responses (e.g., drug metabolizing enzymes, drug transporters, and drug receptors).^14^ These genes are sometimes referred to as pharmacogenes. Other basic pharmacogenetic nomenclature with which healthcare providers should be familiar is shown in Table 1. Genetic variants can result in proteins with normal (i.e., population average), increased, decreased, or complete loss of function/expression. Specific examples of the clinical pharmacogenetic effects on cardiovascular drugs will be reviewed in more detail later on in this tutorial. However, in general, when patients have a genetic variant that results in decreased function of a drug metabolizing enzyme or transporter, then those patients can be exposed to higher drug concentrations systemically (at equivalent doses). When patients have a genetic variant that results in an increase in the function of a drug metabolizing enzyme or transporter, then those patients can be exposed to lower drug concentrations systemically (at equivalent doses). The clinical effects (i.e., increased toxicity or decreased efficacy) of these differences in systemic concentrations depends on whether the drug is activated or deactivated by metabolism. Some drugs, like clopidogrel, are prodrugs because they are activated by metabolism *in vivo*. Thus, the genetic variants would have the opposite clinical effects as typical drugs that are inactivated by metabolism. When patients have a genetic variant in the drug’s target or receptor, then that could alter the receptor’s sensitivity to the drug and therefore affect the drug’s efficacy as well. Pharmacogenetic variants are quite common in the general population. Over 90% of patients carry at least one actionable pharmacogenetic variant, which is defined as a variant with clinically significant effects on risk of toxicity and/or therapeutic failure.^15–17^

The term “pharmacogenetics” was introduced around the end of the 1950s. A 1958 study showed significantly different blood concentrations of the antibiotic isoniazid in European and East Asian patients.^18^ This was attributed to genetic variations in the drug’s metabolism (variants in the N-acetyltransferase 2 gene). The term “pharmacogenomics” was coined more recently.^19^ By the early 1990s, after decades of improvement in analytical methods and advances in our understanding of the human genome, the routine use of clinical pharmacogenetic testing was beginning to be supported.^20^ During the past decade, clinical implementation of pharmacogenetics has significantly advanced, potentially in large part due to the development of clinical practice guidelines that provide evidence-based pharmacogenetic test result interpretations and recommendations for select gene-drug pairs.^21^

Today, pharmacogenetic evidence has reached almost every therapeutic area (e.g., oncology, cardiology, neurology, psychiatry, gastroenterology, and infectious diseases). Although some academic medical centers have spearheaded clinical implementation efforts, other health systems are gradually adopting the practice. Initial pharmacogenetic clinical implementation programs used reactive pharmacogenetic testing,^22^ but the trend has begun to shift mostly to pre-emptive pharmacogenetics testing in the last decade because of increased recognition of the advantages and feasibility of the latter strategy.^23,24^ Reactive pharmacogenetic testing refers to genotyping performed after a decision to prescribe a drug is made or to explain an unexpected drug response. These tests usually focus on the single gene specific for that drug.^24^ Pre-emptive pharmacogenetic testing, on the contrary, occurs prior to any prescribing decisions.^24^ In this scenario, patients are genotyped for a panel of multiple pharmacogenes simultaneously, so that broad pharmacogenetic results are readily available in electronic health records to guide future prescribing. Indeed, the clinical implementation of pre-emptive pharmacogenetics testing in seven health systems across Europe, as part of the Ubiquitous Pharmacogenomics Consortium (U-PGx) program, has demonstrated the feasibility of widespread clinical implementation of pharmacogenetics.^25^ U-PGx has also demonstrated the clinical utility of widespread clinical pharmacogenetics implementation from the highest strength of evidence; in a randomized controlled trial (RCT), pre-emptive genotyping with a panel of 12 pharmacogenes significantly reduced adverse drug events by 30%.^26^

Outside of healthcare systems, direct-to-consumer (DTC) genetic testing companies, such as 23andMe, have expanded their business and modified their practice considerably in the past 2 decades.^27^ Originally, there had been controversy over the ethical practices of DTC genetic testing companies leading to the US Food and Drug Administration (FDA) requiring these companies to discontinue marketing and selling their products until FDA authorization was received.^28^ With the FDA’s continued involvement in its regulation since the 2010s, a new generation of DTC genetic tests have emerged with higher validation requirement and greater separation between clinical (e.g., pharmacogenetic implementation) and nonclinical (e.g., to determine ear-wax phenotype) use.^28,29^ The 23andMe genetic test is Clinical Laboratory Improvement Amendments (CLIA) certified, and thus it can be used to aid clinical decision making. Healthcare providers are increasingly approached by patients to order confirmatory pharmacogenetic testing, interpret results, and recommend follow-up actions.^30,31^ Protections are in place to prevent harm directed toward consumers of DTC genetic testing devices: the Genetic Information Nondiscrimination Act (GINA), which was signed into US federal law in 2008, makes it unlawful to discriminate against individuals based on their genetic test results in regard to health insurance and employment (https://www.eeoc.gov/statutes/genetic-information-nondiscrimination-act-2008). Healthcare providers should be aware that protections conferred by GINA do not extend to certain insurance types (i.e., life, long-term care, and disability) or employers (i.e., employers with fewer than 15 employees and the US military).^32^ However, some states have passed laws that provide additional protection beyond GINA,^33^ and there have not been any GINA violations related to pharmacogenetic test information, to our knowledge.

## STEP 2: GAIN FOUNDATIONAL KNOWLEDGE OF CARDIOVASCULAR PHARMACOGEN ETICS

Pharmacogenetics has received a great deal of attention specifically in oncology where patients are frequently prescribed individualized treatment based on the molecular makeup of their tumor. Cancer at times has been considered the “clear choice” as the disease state to prioritize in precision medicine with the perceived most immediate impact.^34^ However, cardiovascular pharmacogenetics is rapidly gaining popularity as well. Ongoing research in this field spans across multiple cardiovascular drugs/therapeutic classes and across the full scientific spectrum, from discovery through clinical implementation. To date, clinical implementation of pharmacogenetics in the cardiovascular domain has largely centered around three drugs/therapeutic classes: clopidogrel, warfarin, and statins.^35^ These three examples will be covered in step 4 of this tutorial in detail.

There are several common myths and misconceptions concerning cardiovascular pharmacogenetics among healthcare providers today. The most common are summarized and compared with the current reality and evidence in Table 2 and described in more detail below:

### Myth #1: There are no prospective RCTs showing that pharmacogenetics significantly improves clinical outcomes for patients treated with cardiovascular drugs.

Reality: The gold standard for determining the clinical utility of a new therapeutic intervention is to compare clinical outcomes in patients randomized to the new intervention vs. patients randomized to the current standard of care (although many argue that other kinds of clinical evidence are necessary for pharmacogenetics).^36^ Therefore, initial reluctance to use pharmacogenetics for cardiovascular drugs was supported by a lack of prospective RCTs demonstrating clinical utility of pharmacogenetics for cardiovascular drugs. However, more than 20 published RCTs have now shown that pharmacogenetic-guided therapy with cardiovascular drugs significantly improves patient outcomes compared with standard of care.^36^ Three landmark RCT examples include the POPular Genetics trial, in which pharmacogenetic-guided antiplatelet therapy was shown to have superior safety (and similar efficacy) as standard treatment^37^; the EU-PACT trial, which showed that pharmacogenetic-guided warfarin therapy significantly improved time within therapeutic range^38^; and the GIFT trial, which demonstrated that pharmacogenetic-guided warfarin dosing significantly improved both bleeding and thromboembolic outcomes in older patients undergoing elective hip or knee arthroplasty.^39^ These trials and others are thoroughly discussed in later sections. Notably, even though there are now many RCTs to support the clinical utility of cardiovascular pharmacogenetics, it is important to mention the concept of “genetic exceptionalism.” Genetic exceptionalism is a concept in which some argue that genetics is being held to a higher (and unfair) standard compared with other kinds of clinical interventions.^40^ Like other commonly used interventions related to drug therapy, such as adjusting drug therapy based on potential drug–drug interactions, cardiovascular pharmacogenetics is also strongly supported by types of evidence other than RCTs, such as pharmacokinetic studies and pragmatic clinical trials.^36^ Thus, some have argued that RCTs may not even be necessary to support the clinical implementation of every pharmacogenetic test.^36^

### Myth #2: Pharmacogenetics is more important for other kinds of drugs than cardiovascular drugs.

Reality: In a detailed evaluation of pharmacogenetic clinical implementation programs at seven early adopting healthcare systems, the only pharmacogenetic test that was implemented at all seven of the health systems was for a cardiovascular drug (i.e., *CYP2C19* and clopidogrel).^22^ Three cardiovascular drugs (clopidogrel, simvastatin, and warfarin) were among the most commonly implemented pharmacogenetic gene-drug pairs.^22^ In a more recent review of 19 clinical pharmacogenetic implementation programs, most programs targeted implementation in general practice, but the most common specialty practice in which pharmacogenetics was implemented was in cardiology.^41^ Finally, even when a pharmacogenetic test is ordered for a non-cardiovascular drug, due to progress with pre-emptive pharmacogenetic testing, as described above, or pharmacogenetic testing for non-cardiovascular drugs, that genetic test result could still be available in the patient’s electronic health record and have implications for cardiovascular drugs. For example, *CYP2C19* testing is included in commonly used pharmacogenetic tests used in psychiatry,^42^ and those *CYP2C19* test results would apply to clopidogrel as well.

### Myth #3: I do not need to learn about pharmacogenetics because it has not been implemented in my health system.

Reality: Healthcare providers still need to learn about pharmacogenetics because millions of patients now have access to their pharmacogenetic test results through DTC genetic testing. Unlike most types of clinical genetic testing, which requires a prescription from a healthcare provider, patients can now obtain DTC genetic testing without a prescription, and it can include pharmacogenetic results. For example, over 10 million individuals have received DTC genetic testing through 23andMe.^27^ As stated above, the FDA has determined that certain types of pharmacogenetic testing no longer require confirmatory clinical testing.

### Myth #4: Pharmacogenetic tests are too expensive and not reimbursed by third-party payers.

Reality: Due to recent advances in genomic technology, the cost of genetic testing is declining rapidly. Pharmacogenetic tests can now cost as low as $72,^43^ which is similar to and sometimes even lower than other routinely performed clinical tests, such as liver enzyme tests.^44,45^ Moreover, patients’ DNA does not change. Therefore, pharmacogenetic testing may require only one test that can be used lifelong. Most pharmacogenetic testing has been determined to be cost-effective or cost-saving.^43^ The University of Florida’s pharmacogenetic implementation program reported that seven different third-party payers, including Medicare, reimbursed for the *CYP2C19* pharmacogenetic test for clopidogrel.^46^ In the first month of the program, the reimbursement rate was 85% for outpatient claims. A major, recent milestone for pharmacogenetic test reimbursement is expanded coverage for Medicare patients through Molecular Diagnostic Services local coverage determinations.^47^ It states “pharmacogenetics tests are indicated when medications are being considered for use (or already being administered) that are medically necessary, appropriate, and approved for use in the patient’s condition and are known to have a gene(s)-drug interaction that has been demonstrated to be clinically actionable as defined by the FDA (pharmacogenetics information required for safe drug administration) or CPIC guidelines.”^47^ This translates to coverage for 101 drugs to date, including clopidogrel (*CYP2C19*) and statins (*SLCO1B1*). Notably, pharmacogenetic testing for warfarin is currently not covered by Medicare except in rare circumstances (e.g., enrolled in a prospective RCT) based on a national coverage determination from 2010.

### Myth #5: It takes too long to receive pharmacogenetic test results to be clinically useful.

Reality: Even with the aforementioned DTC pharmacogenetic testing becoming more common, patients’ genotypes are usually not known in most clinical scenarios today, and thus a pharmacogenetic test must be ordered if the healthcare provider would like to use pharmacogenetic information. In some clinical situations, a rapid turnaround time (e.g., results the same day) for the genetic test may not be critical. In acute care situations, healthcare providers are often concerned that the turnaround times for pharmacogenetic test results will be too long to be clinically useful. However, health systems that implemented reactive pharmacogenetic testing reported a turnaround time for pharmacogenetic test results in as little as a few hours and a median turnaround time of 2.6 days.^22^ Furthermore, pre-emptive pharmacogenetic testing, as described above, is becoming a more common solution. Moreover, a commercial company has developed a rapid point-of-care pharmacogenetic test for *CYP2C19* (granted FDA 510k clearance in March 2023), which can be performed at the patients’ bedside directly from a buccal swab and provide results in as little as 1–2 hours.^48^ In the RAPID GENE trial,^49^ patients undergoing percutaneous coronary intervention (PCI) for acute coronary syndrome or stable angina were randomly assigned to rapid point-of-care pharmacogenetic testing for CYP2C19*2 or the standard of care. Patients randomized to the point-of-care pharmacogenetic testing group had significantly lower rates of high on-treatment platelet reactivity than those assigned to the standard of care (*P* = 0.009).

## STEP 3: LEARN THE DIFFERENT ORGANIZATIONS THAT RELEASE CARDIOVASCULAR PHARMACOGENETIC GUIDELINES AND RECOMMENDATIONS

Because of the extremely large and rapidly growing body of literature, it is impossible to cover all of the knowledge on the use of cardiovascular pharmacogenetics that healthcare providers need in a single tutorial. Therefore, it is important for healthcare providers to be familiar with the different resources that provide clinical pharmacogenetic recommendations, and the strengths and limitations of those different resources. The Clinical Pharmacogenetics Implementation Consortium (CPIC) was created in 2009 as a joint effort between the NIH and the Pharmacogenomics Knowledge Base (PharmGKB), and has published 26 guidelines as of late 2022.^21^ PharmGKB also includes the drug label annotations from regulatory agencies, such as the FDA and the European Medicines Agency (EMA) for interpretations and/or actions required for specific genotypes.^50^ To optimize the clinical utility of pharmacogenetics, the NIH-funded Implementing GeNomics In practice network (IGNITE) was established in 2013.^51^ Its initial iteration included multiple site projects aiming to test implementation models to enhance the framework for translating genomic medicine into practice. The IGNITE Pragmatic Trials Network was formed in 2018 to examine the clinical and cost effectiveness of genomic medicine approaches.^52^ Outside the United States, many professional societies have also been publishing guidelines for the past several years. The Dutch Pharmacogenetics Working Group (DPWG) – founded by the Royal Dutch Pharmacists Association in 2005 – is the most widely recognized of these, having published 47 guidelines as of late 2022.^53^ Pharmacogenetic guidelines are also published by other organizations (e.g., Canadian Pharmacogenomics Network for Drug Safety (CPNDS)), but discussion of these guidelines is outside the scope of this tutorial. This tutorial focuses on the three most commonly used guidelines and resources for cardiovascular drugs used by healthcare providers in the United States: the CPIC, the FDA, and the American Heart Association/ American College of Cardiology (AHA/ACC). Table 3 summarizes the clinical pharmacogenetic recommendations from the CPIC, the FDA, and the AHA/ACC for the three most commonly implemented cardiovascular drugs and drug/classes^22^: clopidogrel, statins, and warfarin.

### Clinical Pharmacogenetics Implementation Consortium

The CPIC represents a consortium of scientists and clinicians who are experts in pharmacogenetics as well as its clinical implementation (https://cpicpgx.org/). One barrier to implementation of pharmacogenetic testing in the clinic is the difficulty in translating genetic laboratory test results into actionable prescribing decisions for affected drugs. The CPIC’s goal is to address this barrier to clinical implementation of pharmacogenetic tests by creating, curating, and posting freely available, peer-reviewed, evidence-based, updatable, and detailed gene/drug clinical practice guidelines.^21^ The CPIC guidelines do not cover whether or not a pharmacogenetic test should be ordered. The CPIC classifies gene-drug associations by strength of evidence (ordered A-D with A having the strongest level of evidence). Associations classified into levels A and B meet the threshold for clinical action (i.e., a therapeutic regimen can be changed based on genotype). Just as important, the CPIC assigns levels of C or D for those gene-drug pairs where the evidence is weak and should not be used to guide prescribing. This is especially important because some commercially available genetic tests may provide test results for drugs with level C or D evidence along with drugs with level A or B evidence. The CPIC website can be searched by gene or drug and provides links to corresponding guidelines. Importantly, the CPIC guidelines for individual gene-drug pairs are periodically updated as more evidence becomes available. Moreover, the guidelines provide tangible implementation resources, such as clinical decision support language, to enable integration of pharmacogenetic test results into the electronic health record. There are currently CPIC guidelines for three drugs/drug classes used to treat cardiovascular diseases: clopidogrel,^54^ statins,^55^ and warfarin.^56^ We recommend the CPIC guidelines as the most comprehensive and primary resource for any detailed information beyond the scope of this introductory tutorial for healthcare providers practicing in the United States for a few reasons. First, the CPIC consists of experts specializing specifically in pharmacogenetics. Second, the CPIC uses a standardized and transparent process for pharmacogenetic evidence evaluation and making recommendations. Third, only the CPIC provides the detailed information on the specific alleles, diplotypes, and phenotypes necessary for translation of the recommendations into clinical practice. Fourth, the other two organizations covered next (FDA and AHA/ACC), can have inconsistencies in their pharmacogenetic information that can be confusing for healthcare providers^57^ (i.e., the FDA provides different pharmacogenetic information in the Table of Pharmacogenomic Associations vs. the Table of Pharmacogenomic Biomarkers in Drug Labeling, and the AHA/ACC provides different pharmacogenetic information in guidelines vs. statements).

### US Food and Drug Administration

The FDA website houses the routinely updated Tables of Pharmacogenomic Associations and Pharmacogenomic Biomarkers in Drug Labeling (https://www.fda.gov/drugs/science-and-research-drugs/table-pharmacogenomic-biomarkers-drug-labeling). The “Associations” table publishes gene-drug pairs that, as deemed by the FDA, are supported by sufficient evidence, whereas the “Biomarkers” table conveniently presents all pharmacogenomic information contained in FDA drug labels. The “Associations” Table is divided into three sections. Section 1 contains gene-drug pairs with evidence to support clinical recommendations, whereas sections 2 and 3 list pharmacogenomics associations that indicate a potential impact on drug safety or pharmacokinetics, respectively. As of late 2022, 11 cardiovascular drugs are listed throughout the table, 3 of which are included in section 1 (clopidogrel, propafenone, and warfarin). The “Biomarkers” Table shows that 17 cardiovascular drugs (including rosuvastatin) currently have pharmacogenomics information provided in their drug label. However, not all drugs listed in the “Biomarkers” Table are also contained in the “Associations” Table, and vice versa. Additionally, some pharmacogenomic information in drug labels may be outdated. For example, the warfarin label only provides dosing recommendations based on CYP2C9 *2 and *3 and does not acknowledge the additional alleles important in other ethnic groups (see warfarin section below). Moreover, even if a drug is listed in the “Biomarkers” Table, that does not necessarily mean that clinical action is recommended by the FDA. For example, rosuvastatin and *SLCO1B1* are listed in the “Biomarkers” Table, but the mention of pharmacogenetics is only informational, and no clinical action is recommended in the rosuvastatin label (i.e., “The impact of this polymorphism [*SLCO1B1*] on efficacy and/or safety of rosuvastatin has not been clearly established”). Due to rather vague therapeutic recommendations provided in cardiovascular drug labels, inconsistencies between the FDA tables, and lack of citations accompanying clinical study references, these FDA tables may be considered a secondary resource useful in instances where a CPIC guideline is unavailable for the gene-drug pair in question (e.g., we would recommend the FDA tables for propafenone, which is not covered by CPIC guidelines).

### American Heart Association/ American College of Cardiology

The AHA and the ACC are the leading resources in the United States for clinical practice recommendations for cardiovascular diseases, but neither have a centralized repository for pharmacogenetic recommendations or guidelines. This is likely because neither provides much pharmacogenetic guidance in general. As of late 2022, keyword searches for “pharmacogenetics” and “pharmacogenomics” at the Library of Guidelines and Clinical Documents on the ACC website (https://www.acc.org/guidelines) and the Guidelines and Statements Search on the AHA website (https://professional.heart.org/en/guidelines-and-statements/guidelines-and-statements-search) yield very limited results. The ACC *Recommendations for Beta Blockers and Amiodarone in Patients Undergoing CABG* from 2021 states, “Beta-blocker pharmacogenetic variation may have a role” in mortality rates after coronary artery bypass graft surgery. When filtering the “pharmacogenetics” or “pharmacogenomics” AHA search to only “Clinical Practice Guideline” document types, three articles are returned but only the *Guidelines for the primary prevention of stroke* (2014) has guidance, stating “Pharmacogenetic dosing of vitamin K antagonists may be considered when therapy is initiated.” Although there is a paucity of guidelines with pharmacogenetic recommendations from these organizations, the AHA released a scientific statement in 2016 stating, There are specific drugs for which pharmacogenetic variant information can be reasonably used today, and refers readers to the FDA pharmacogenetic tables, PharmGKB, and CPIC.^58^ However, unlike guidelines, scientific statements from the AHA do not undergo the same rigorous and transparent process for evidence evaluation, and clinical recommendations are technically not allowed in AHA scientific statements. Due to the lack of a centralized resource for pharmacogenetic-specific recommendations, outdated content, and difficulty searching/retrieving the documents that are available, we recommend that pharmaco-genetic guidelines or statements from AHA/ACC should (along with FDA material) also be considered only as a secondary resource to CPIC.

## STEP 4: KNOW THE CURRENT CARDIOVASCULAR THERAPIES TO FOCUS ON CLINICALLY AND THE SUPPORTING EVIDENCE

### Clopidogrel

The most ordered pharmacogenetic test for cardiovascular drugs in clinical practice is *CYP2C19* to predict clopidogrel response.^22^ Clopidogrel is a pro-drug metabolized to its active form by the CYP2C19 enzyme. The CPIC recommends with level A evidence that clopidogrel be avoided in individuals with a poor or intermediate CYP2C19 metabolizer phenotype using an alternative P2Y_12_ inhibitor (e.g., ticagrelor or prasugrel) instead and that clopidogrel can be considered in individuals with a normal, rapid, or ultrarapid CYP2C19 metabolizer phenotype (Table 3).^54^ Results from RCTs and meta-analyses have guided these CPIC recommendations.^59^ In POPular Genetics, a genotype-guided strategy (ticagrelor or prasugrel in all except normal, rapid, or ultrarapid CYP2C19 metabolizers who were de-escalated to clopidogrel) was noninferior to standard-treatment (ticagrelor or prasugrel in all) for the primary composite outcome of death, myocardial infarction (MI), stent thrombosis, stroke, or major bleeding at 12 months (5.1% vs. 5.9%, respectively; noninferiority *P* < 0.001) and superior to standard-treatment in reducing the incidence for the primary bleeding outcome (9.9% vs. 12.5%, respectively, *P* = 0.04) in patients undergoing PCI.^37^ In TAILOR-PCI, the primary outcome of composite cardiovascular death, MI, stroke, stent thrombosis, and severe recurrent ischemia events at 12 months occurred in 4% of participants randomized to the genotype-guided arm (clopidogrel in all except poor or intermediate CYP2C19 metabolizers who were escalated to prasugrel or ticagrelor) and 5.9% of participants (hazard ratio (HR): 0.66, *P* = 0.06) randomized to the standard-treatment arm (clopidogrel in all).^60^ Of note, the overall event rate of the trial was lower than expected impacting the power to detect this 34% relative risk reduction effect. A *post hoc* analysis of these data showed that this nominal effect may have been driven by a potential benefit of the genotype-guided approach in the first 3 months after PCI (HR: 0.21, *P* = 0.001).^60^ A meta-analysis of 7 RCTs (15,949 patients) further validates the importance of *CYP2C19* genotype status by showing that the benefit of prasugrel or ticagrelor over clopidogrel was primarily due to variation in this pharmacogene.^61^

Clopidogrel is also used to prevent recurrent stroke in patients with transient ischemic attack or acute ischemic stroke.^54^ A meta-analysis of encompassing 15 studies in 4,762 patients treated with clopidogrel, showed that the *CYP2C19* no function allele carriers had a higher risk of stroke compared with noncarriers (12.0% vs. 5.8%; risk ratio: 1.92, 95% confidence interval (CI): 1.57–2.35, *P* < 0.001).^62^ Most recently, a prospective, randomized, double-blind, placebo-controlled trial of ticagrelor vs. clopidogrel, the CHANCE-2 trial (*n* = 6,412), conducted in *CYP2C19* no function allele carriers only, showed a lower risk of subsequent stroke or transient ischemic attack when treated with ticagrelor (6.0% vs. 7.6%, HR: 0.77, 95% CI: 0.64–0.94, *P* = 0.008).^63^ These results suggest that prospective *CYP2C19* testing in patient with neurovascular disease is beneficial.

### Statins

Statins are mostly well-tolerated, although some patients report statin associated musculoskeletal symptoms (SAMS). The SAMS refers to a range of disorders from the rare rhabdomyolysis (1 in 10,000) to muscle pain with evidence of muscle damage or myopathy (1 in 2,000) to muscle symptoms without evidence of damage or myalgia (up to 1 in 10).^55^ The *SLCO1B1* gene encoding the hepatic transporter OATP1B1 is the most validated gene underlying the risk for statin myopathy. First identified through a genomewide association study of myopathy in patients treated with simvastatin 80 mg,^64^ the reduced function haplotype (*5) has since been extended to other SAMS phenotypes^65^ and statins.^66^ Beyond symptoms, the *5 variant has also been associated with premature statin discontinuation for simvastatin^67^ and atorvastatin.^68^ Importantly, SAMS risk (in *5 allele carriers) appears to be a class effect pharmacokinetically, although the strength of association is statin type- and dose-dependent.^65^ Additionally, there is recent evidence of a differential effect of *SLCO1B1* genotype by sex requiring further investigation.^69^ The biggest updates to the most recent CPIC guidelines for statins (published in 2022), compared with the 2014 update, is the expansion of statin types and genotypes beyond only simvastatin and *SLCO1B1*, respectively (Table 3).^55,70^ Designed to mirror the 2018 multi-society guidelines for managing blood lipids,^71^ the 2022 CPIC guidelines for statins begin with an assessment of the patient’s cardiovascular risk and desired statin lipid lowering intensity (low, moderate, or high). Based on this assessment and the patient’s SLCO1B1 phenotype (reduced or poor function), the guidelines provide statin types/doses and their associated SAMS risk. For example, a provider assessing a patient who is an *SLCO1B1* reduced function carrier with established atherosclerotic cardiovascular disease requiring high-intensity statin therapy should avoid atorvastatin 80 mg because of its high risk of SAMS. Instead, rosuvastatin 20 mg would be preferred to achieve a similar LDL-C lowering effect. The statin CPIC guidelines are not intended to replace the 2018 multi-society guidelines.^55^ Beyond *SLCO1B1*, there is evidence that genetic variation in *CYP2C9* is associated with increased exposure to fluvastatin.^72^ Moreover, variation in *ABCG2*, which encodes an efflux transporter (BCRP) that modulates the absorption and disposition of rosuvastatin, is associated with rosuvastatin exposure.^73^ Accordingly, the CPIC guidelines include recommendations for dosing rosuvastatin based on *ABCG2* phenotype in combination with *SLCO1B1*, and for dosing fluvastatin based on *CYP2C9* phenotype. With this recent expansion of CPIC guidelines to commonly prescribed high-intensity statins (i.e., atorvastatin and rosuvastatin), we anticipate increased use of pharmacogenetic-guided statin selection and dosing in clinical care. The CPIC guideline recommendations are based on level A strength of evidence.^55^ Additionally, the FDA has recently determined that clinical confirmatory testing is no longer required for the 23andMe DTC pharmacogenetic test of *SLCO1B1*-simvastatin.^74^

### Warfarin

The 2017 CPIC guidelines for genotype-guided warfarin therapy focuses on dose requirements using validated published pharmacogenetic algorithms based on *CYP2C9*, *VKORC1*, *CYP4F2*, and rs12777823 genotype (level A evidence; Table 3). Variants in the *CYP2C9* and *VKORC1* genes contribute to decreased warfarin metabolism and altered sensitivity, and patients with these polymorphisms may need modified starting doses to achieve the desired anticoagulant effects.^56^ Three large RCTs (COAG, EU-PACT, and GIFT) have examined the efficacy of genotype-guided warfarin dosing compared with either a fixed (e.g., 5 mg/day) or a clinically guided dosing strategy.^38,39,75^ The COAG and EU-PACT trials predominately included patients with atrial fibrillation or venous thromboembolism and had the primary outcome of time in therapeutic international normalized ratio (INR) range (usually the therapeutic range is 2–3) in the initial 4–12 weeks of warfarin therapy.^38,75^ The GIFT trial included patients undergoing hip or knee arthroplasty and had the primary composite outcome of major bleeding, INR ≥ 4, venous thromboembolism, or death.^39^ Both EU-PACT and GIFT showed improved outcomes with genotype-guided dosing, whereas COAG found no difference between a genotype-guided vs. clinically guided dosing approach. The comparator groups in the EU-PACT and COAG were different and likely contributed to the disparate results. The EU-PACT comparator arm used a fixed dose warfarin regimen for the first 3 days (i.e., either 10 or 5 mg depending on age) and COAG used a clinically guided dosing strategy that considered variables, such as age, race, drug interactions, and early INR values, to select the warfarin dose. Unlike the EU-PACT and GIFT trials, in which > 90% of participants were of European ancestry, 27% of COAG participants were African American. Genotyping across trials was limited to testing for the *VKORC1*-1639G>A and CYP2C9*2 and *3 variants (plus CYP4F2*3 in GIFT), which are primary variants influencing warfarin dose in European ancestry patients.^56^ Additional *CYP2C9* variants, namely the *5, *6, *8, and *11 alleles, reduce warfarin dose requirements and are collectively more prevalent that the *2 and *3 alleles combined in those of African ancestry.^76^ Neglecting to test for these additional *CYP2C9* alleles is associated with overdosing of warfarin in African Americans, as was observed in the COAG trial.^75,77^ Based on these data, the CPIC guidelines recommend that genotype only be used to dose warfarin in persons of African ancestry when genotyping includes testing for the CYP2C9*5, *6, *8, and *11 alleles.^56^ Free online tools are publicly available to assist healthcare providers with the use of warfarin pharmacogenetics (e.g., warfarindosing.org).

## STEP 5: DISCUSS AN EXAMPLE PATIENT CASE OF CLINICAL CARDIOVASCULAR PHARMACOGENETICS

JT is a 67-year-old patient (pronouns: he/him/his) presenting to the hospital for emergency reperfusion following an ST-elevation myocardial infarction (STEMI). He first became symptomatic ~60 minutes prior to presentation and was transported to the hospital via ambulance by emergency medical services who administered 325 mg of chewable aspirin. JT has a medical history that includes longstanding hypertension (untreated) and obesity (body mass index of 30.9) as well as recent type 2 diabetes mellitus (diagnosed 1 year ago) for which he takes metformin 1 gram twice daily. He is insured under Medicare but does not have separate prescription drug coverage. Following a loading dose of ticagrelor (180 mg), he undergoes emergency coronary angiography and PCI of a 99% proximal-mid stenosis in the left anterior descending artery for his STEMI. After the procedure, he is started on appropriate medical therapy with aspirin 81 mg daily, ticagrelor 90 mg twice daily, atorvastatin 80 mg daily, metoprolol XL 100 mg daily, and lisinopril 10 mg daily. An echocardiogram shows a left ventricular ejection fraction of 45%. Prior to discharge, JT’s cardiologist discovers that JT was pre-emptively genotyped for a panel of variants among a handful of pharmacogenes and that information is already available in the electronic health records. As *CYP2C19* genotype can be used to optimize antiplatelet therapy, JT’s cardiologist searches the genotype results for this pharmacogene. Test results reveal normal metabolizer phenotype status (CYP2C19*1/*1 genotype). JT’s cardiologist also observes the *SLCO1B1* results, which reveal that JT carries one copy of the *5 variant.

The antiplatelet pharmacotherapeutic options are between high potency agents (i.e., prasugrel and ticagrelor) and clopidogrel. Clinical decisions for these therapies are based on goals to maximize ischemic protection while limiting risk of bleeding. Based on clinical RCT data, for which *CYP2C19* genotype was not considered, the current ACC/AHA dual antiplatelet therapy guidelines recommend (class IIa recommendation, level of evidence B) ticagrelor or prasugrel over clopidogrel for patients who are treated with PCI for an acute coronary syndrome.^78^ However, a strategy of *CYP2C19* genotype-guided antiplatelet prescribing with de-escalation to standard-dose clopidogrel (75 mg daily) for patients having normal or ultrarapid metabolizer phenotypes has been shown in an RCT to be noninferior to the standard use of the more potent P2Y_12_ inhibitors in terms of ischemic event protection with a lower risk of bleeding.^37^ Accordingly, the most updated CPIC guidelines recommend with “strong” classification that standard dose (75 mg daily) can be used in normal metabolizers specifically when clopidogrel is already being considered for clinical use.^54^ Thus, although it would be fine for JT to be continued with ticagrelor on discharge, de-escalation to clopidogrel from ticagrelor would also be reasonable considering JT’s *CYP2C19* genotype, especially if bleeding risk and cost are concerns. Results from the TROPICAL ACS trial also support a biomarker-driven de-escalation strategy for antiplatelet therapy in the setting of acute coronary syndrome.^79^

It is important to consider routine clinical factors in addition to genetics when selecting a P2Y_12_ inhibitor. One method is the Age, Body Mass Index, Chronic Kidney Disease, Diabetes, and Genotyping (ABCD-GENE) score, which predicts the risk of ineffective clopidogrel therapy in individual patients.^80^ A recent *post hoc* analysis of TAILOR-PCI identified that, among patients prescribed clopidogrel, a high ABCD-GENE score (≥ 10) was associated with an increased risk of death, MI, or stroke compared with a low score (5.2% vs. 2.6%, HR: 2.04, 95% CI: 1.35–3.07, *P* < 0.001).^81^ In this case example, patient JT has an ABCD-GENE score of 7 (3 points for diabetes +4 points for obesity) suggesting that he may not be at increased risk of clopidogrel failure. This new information concerning JT’s risk of a subsequent ischemic event (in addition to the clinical details and *CYP2C19* genotype) serves to reinforce that it would be reasonable to discontinue ticagrelor and start clopidogrel 75 mg daily instead (i.e., to implement a genotype-based de-escalation strategy) prior to discharge.

JT’s cardiologist also inspected the *SLCO1B1* results, which are relevant to the atorvastatin 80 mg daily regimen that JT was prescribed following his STEMI. This is appropriate therapy per the 2018 multi-society cholesterol treatment guidelines,^71^ which recommend a high-intensity LDL-C lowering statin as first-line treatment for atherosclerotic cardiovascular disease with the highest strength of evidence. As previously stated, the 2022 CPIC statin guidelines are designed to be used in conjunction with, rather than replace, the cholesterol guidelines. Thus, the CPIC guidelines never make recommendations to discontinue or avoid statins when they are indicated based on the cholesterol guidelines. Instead, they provide guidance on changing statin type or dose that optimizes efficacy and safety. In the clinical example, JT’s *SLCO1B1* diplotype (*1/*5) puts him at high risk for SAMS if receiving atorvastatin 80 mg daily. The CPIC suggests that rosuvastatin 20 mg daily as an alternative would put JT at lower risk for SAMS while providing a similar LDL-C lowering statin intensity as atorvastatin 80 mg daily. Of course, *ABCG2* genotype would provide an additional clinical clue that can further guide statin therapy if that information is available (the CPIC recommends considering rosuvastatin ≤ 10 mg daily for ABCG2 poor function). With *ABCG2* test results unavailable, rosuvastatin 20 mg daily remains a reasonable alternative to atorvastatin based on SLCO1B1*1/*5.

In the above clinical scenario, *CYP2C19* and *SLCO1B1* genotype were fortunately available in the patient’s health record preemptively. However, this is currently the exception rather than the norm. Incorporation of pharmacogenetic test results into standard practice can benefit from a coordinated effort from the patient’s hospital or health system. Lessons learned from early adopting institutions include streamlining test ordering by including genotype information alongside other routine laboratory tests and providing support for result interpretation.^82^ This clinical support often includes pharmacist consultations or interruptive electronic clinical decisions support alerts. Clinical decisions support alerts can be designed to trigger only if the genotype result suggests a change in a drug order. Engagement with cardiology stakeholders is helpful to determine the desired level of support. Local practices can also inform what strategies may best fit local prescribing practices (e.g., for *CYP2C19* results, an escalation strategy may be best if clopidogrel is standard; de-escalation if ticagrelor or prasugrel are standard).

## STEP 6: DEVELOP AN APPRECIATION FOR EMERGING AREAS IN CARDIOVASCULAR PHARMACOGENETICS

This tutorial mainly focused on clopidogrel, statins, and warfarin because those are the three cardiovascular drug/drug classes for which pharmacogenetic testing is most commonly implemented in clinical practice today.^22^ However, newer advances in the field are rapidly emerging, such as for other cardiovascular drug classes (e.g., beta-blockers) and approaches (e.g., polygenic risk scores). Therefore, current healthcare providers and students should develop an appreciation for these emerging areas because they will probably be clinically implemented in the near future.

The next cardiovascular drug class that will most likely have more widespread clinical implementation in the near future is beta-blockers. Carvedilol, metoprolol, nebivolol, and propranolol are all metabolized by *CYP2D6*. The *CYP2D6* gene is highly polymorphic, meaning that there are many genetic variants in *CYP2D6* among humans. This results in a large amount of heterogeneity in CYP2D6 enzyme activity and hence a large amount of heterogeneity in the metabolism of those four beta-blockers among patients. For example, people with predominant East Asian and Oceanian ancestry have the lowest global prevalence of CYP2D6 poor metabolizers, whereas people with predominant European ancestry have the highest global proportion.^83^ Patients who are CYP2D6 poor metabolizers have higher systemic concentrations of those 4 beta-blockers, and thus those patients may require lower doses (or more careful dose titration) with those 4 beta-blockers. The FDA Table of Pharmacogenomic Biomarkers in Drug Labeling and the FDA Table of Pharmacogenetic Associations both already include pharmacogenetic information for those 4 beta-blockers and *CYP2D6*. The CPIC is currently writing a clinical practice guideline for beta-blocker pharmacogenetics, which is expected to be published by the end of 2023. To our knowledge, the AHA/ACC have not published any clinical recommendations based on *CYP2D6* and beta-blockers.

Most of the current pharmacogenetic recommendations for cardiovascular drugs are based on one or a few candidate pharmacogenes not spanning the genome (such as statins with candidate genes *SLC01B1*, *ABCG2*, and *CYP2C9*). However, like most other complex clinical traits, patient responses to cardiovascular drugs are polygenic, meaning that multiple genes across the full genome are involved. Polygenic risk scores are a newer approach that combine the effects of multiple genomewide variants into a single score. Polygenic risk scores are being used to predict patients’ risks of developing cardiovascular diseases^84^; recent evidence also supports their use for predicting patients’ responses to cardiovascular drugs, such as statins,^85–87^ beta-blockers,^88,89^ and proprotein convertase subtilisin/kexin type 9 (PCSK9) inhibitors.^90^ For example, a polygenic risk score successfully predicted patients’ with heart failure long-term survival benefit from beta-blockers in four different datasets. However, healthcare providers should be aware of the current limitations of polygenic risk scores. Most polygenic risk scores only apply to patients with European ancestry, and they still need prospective validation before they should be used clinically. Currently, there are no clinical pharmacogenetic guidelines that recommend with strong evidence the implementation of polygenic risk scores to optimize cardiovascular therapies for patients.

## CONCLUSION

In summary, pharmacogenetics is becoming more and more important for cardiovascular drugs per evidence-based guidelines and as new data is generated. Millions of Americans are becoming empowered to direct their own cardiovascular drug therapies by obtaining FDA-approved pharmacogenetic test results through DTC genetic testing. To keep up with these advances and move closer toward universal implementation, healthcare providers should be equipped with the necessary cardiovascular pharmacogenetics knowledgebase. Furthermore, healthcare providers should understand the best resources to maintain competence as new pharmacogenetic recommendations become available. Societies publishing clinical pharmacogenetic recommendations for cardiovascular drugs in the United States, such as the CPIC, the FDA, and the AHA/ACC, should work together to resolve inconsistencies in their recommendations, so that healthcare providers can have clear guidance. Societies should also be more proactive in including pharmacogenetic experts on clinical guidelines writing groups even if the overarching focus does not center on pharmacogenetics. On behalf of the PGRN, we developed this tutorial to provide introductory education for healthcare providers on the use of cardiovascular pharmacogenetics in clinical practice. This document was specifically designed for healthcare providers less familiar with the pharmacogenetics. For complex clinical situations, a pharmacogenetics expert will likely be necessary for consultation. However, for clearcut scenarios in which clinical decisions are unequivocally supported by pharmacogenetic evidence, this tutorial will provide healthcare providers with the first steps necessary to perform pharmacogenetic-guided prescribing and counseling for cardiovascular drugs. Importantly, genetic test results represent only one additional set of information that needs to be considered along with other clues (e.g., concomitant therapies, compelling indications, patient preference, insurance coverage, and kidney function) that guide drug selection and dosing. Altogether, the future remains bright for pharmacogenetics and its potential to help reduce the burden of cardiovascular disease in the United States.

## Figures and Tables

**Table 1:** Basic pharmacogenetic nomenclature

	Definitions
Alleles	Alternative forms of a given DNA or genomic sequence that is located at a specific position on a specific chromosome Wild type: Most common or reference allele (“major allele”) Variant: Less common allele (“minor allele”)
Diplotype	A pair of haplotypes where one haplotype is inherited from the mother and one from the father
Gene	Basic unit of inheritance, that occupies a specific position (locus) within a genome or chromosome and contains the information needed to specify a phenotypic trait in an organism
Genetic variation	Differences in the DNA or genomic sequence compared with a reference sequence among individuals or populations, that leads to diversity in the gene pool and differences in population allele frequencies
Genome	Complete set of DNA, including all of its genes, in an individual or cell. The human genome includes DNA in both the nucleus of a cell (nuclear genome) and the mitochondria (while small, the mitochondrial genome has some vital genes and related genetic disorders). The human genome has 23 pairs of chromosomes (diploid organisms) containing 20,000–25,000 genes and 3.2 billion base pairs
Genotype	A pair of alleles at a specific location in the DNA or genome, where one allele is inherited from the mother and one from the father^a^ Homozygous genotype: Two identical alleles of one or more specific genes (e.g., A/A) Heterozygous genotype: Two different alleles of one or more specific genes (e.g., A/B)
Haplotype	A set of genomic variations that tend to be inherited together due to linkage disequilibrium
Mutation	Genetic variation with a frequency of < 1% in the population, typically considered to be pathogenic
Phenotype	An individual’s observable trait or characteristic.Pharmacogenetics examples: Enzyme metabolism: ultra-rapid, rapid, normal, intermediate, and poor metabolizers Pharmacokinetics: plasma drug concentration, AUC, clearance, C_max_ Pharmacodynamics: responder, nonresponder Drug toxicity/ADR: drug-induced skin reactions
Polymorphisms	Genetic variation with a frequency of > 1% in the population, typically considered to be normal variation in the population and are sometimes associated with disease risk or drug response
Star allele	A nomenclature system to classify haplotypes of pharmacogenes by numbers, based on chronological order of discovery. One example is CYP2C19*2, which is read aloud as “CYP2C19-star-two”

ADR, adverse drug reaction; AUC, area under the concentration-time curve; *C*_max_, maximum concentration; DNA, deoxyribonucleic acid.

a There are some important caveats of differences in inheritance patterns, such as for the mitochondrial genome and X inactivation. There are also imprinted loci/parent-of-origin effects, and more complex variation such as Copy Number Variation. Readers are referred to the National Human Genome Research Institute website for more information: https://www.genome.gov/about-genomics.

**Table 2:** Common myths/misconceptions about cardiovascular pharmacogenetics compared with the current evidence

Common cardiovascular pharmacogenetic myths/misconceptions	Current evidence
#1: There are no prospective RCTs showing that pharmacogenetics significantly improves outcomes for patients treated with cardiovascular drugs	More than 20 prospective RCTs now show that pharmacogenetic-guided cardiovascular drug therapy significantly improves patient outcomes compared with non-pharmacogenetic guided cardiovascular drug therapy^36^
#2: Pharmacogenetics is more important for other kinds of drugs than cardiovascular drugs	The most commonly implemented pharmacogenetic tests at early adopting health systems are for cardiovascular drugs,^22^ and the most common specialty in which pharmacogenetic tests are used is cardiology^41^
#3: I do not need to learn about pharmacogenetics because pharmacogenetics has not been implemented in my health system	Healthcare providers still need to learn about pharmacogenetics because millions of patients now have their pharmacogenetic test results through direct-to-consumer genetic testing^27^
#4: Pharmacogenetic tests are too expensive and not reimbursed by third-party payers	Pharmacogenetic tests can now cost as low as $72, and they are usually cost-effective or cost-saving.^43^ Multiple third-party payers, including Medicare, now reimburse for pharmacogenetic tests for cardiovascular drugs at high rates (e.g., 85% reimbursement rate)^46^
#5: It takes too long to receive pharmacogenetic test results to be clinically useful	Pharmacogenetic test results can be returned the same day, or be preemptively stored in the EHR,^22,41^ and point-of-care pharmacogenetic tests have been cleared an/or are under review by the FDA

EHR, electronic health record; FDA, Food and Drug Administration; RCT, randomized controlled trial.

**Table 3:** Summary of cardiovascular pharmacogenetic guidelines and recommendations from select commonly used resources in the United States

Drug (s)	Gene(s)	CPIC^a^	AHA/ACC	FDA
Clopidogrel	*CYP2C19*	CYP2C19 phenotype guided recommendations available for patients undergoing percutaneous coronary intervention in the setting of percutaneous coronary interventions/acute coronary syndromes and in the setting of stroke/TIA.^54^ Alternative antiplatelet agents (e.g., ticagrelor, prasugrel, or ticlopidine) are recommended for patients that are CYP2C19 intermediate or poor metabolizers (depending on the indication)	The routine clinical use of genetic testing to screen patients treated with clopidogrel who are undergoing PCI is not recommended. Class III (no benefit), Level of Evidence: C.^91^ Genetic testing might be considered to identify whether a patient at high risk for poor clinical outcomes is predisposed to inadequate platelet inhibition with clopidogrel. Class IIB, Level of Evidence: C.^91^ When a patient predisposed to inadequate platelet inhibition with clopidogrel is identified by genetic testing, treatment with an alternate P2Y12 inhibitor (e.g., prasugrel or ticagrelor) might be considered. Class IIB, Level of Evidence: C.^91^ *CYP2C19* genotyping in patient on clopidogrel may provide useful prognostic data for cardiovascular risk prediction. Genotyping to escalate therapy in LOF carriers undergoing elective PCI (stable CAD) may be considered in specific clinical scenarios. However, routine genotyping is not recommended in patients with ACS.^92^	Intermediate and poor metabolizers. Results in lower systemic active metabolite concentrations, lower antiplatelet response, and may result in higher cardiovascular risk. Consider use of another platelet P2Y12 inhibitor.^93^ A black box warning appears on the package label for clopidogrel (Plavix)^94^ Effectiveness of clopid depends on conversion to an active metabolite by the cytochrome P450 (CYP) system, principally CYP2C19 Tests are available to identify patients who are CYP2C19 poor metabolizers Consider use of another platelet P2Y12 inhibitor in patients identified as CYP2C19 poor metabolizers
Atorvastatin, fluvastatin, lovastatin, pitavastatin, pravastatin, simvastatin, rosuvastatin	*SLCO1B1* *CYP2C9* *ABCG2*	SLCO1B1 phenotype guided recommendations stratified by desired statin dose intensity and SLCO1B1 phenotype are provided to minimize risk of statin associated myopathy. Dosing recommendations for fluvastatin are also based on CYP2C9 phenotype in addition to SLCO1B1. Dosing recommendations for rosuvastatin are also based on ABCG2 phenotype in addition to SLCO1B1^55^	*SLCO1B1* is mentioned as a factor for statin associated myopathy in a Scientific Statement on statin safety and associated adverse events from the AHA, but no recommendations regarding testing to reduce the risk of myopathy are included^95^ Pharmacogenetic testing is not mentioned in the guidelines for management of blood cholesterol^71^	*SLCO1B1* is mentioned in the table of pharmacogenetic associations for atorvastatin, simvastatin, and rosuvastatin^93^
Warfarin	*CYP2C9*	Genotype guided dosing for warfarin is provided accounting for genotypes for these 4 loci and considering African ancestry^56^	Pharmacogenetic markers for warfarin mentioned in several AHA statements,^58,96^ however, no specific dosing guidelines or testing recommendations based on genotype or indication are provided. The use of algorithms to select the optimal dose of are mentioned.	Dosing table provided in drug label based on CYP2C9 *2/*3 alleles and *VKORC1* genotype^97^
	*CYP4F2*			
	*VKORC1*			
	*CYP2C* cluster variant (rs12777823)			

ACS, acute coronary syndrome; AHA/ACC, American Heart Association/ American College of Cardiology; CAD, coronary artery disease; CPIC, Clinical Pharmacogenetics Implementation Consortium; FDA, US Food and Drug Administration; LOF, loss-of-function; PCI, percutaneous coronary intervention; TIA, transient ischemic attack.

a We recommend the CPIC as the primary resource for pharmacogenetic clinical practice guidelines and recommendations.
